# Blood transcriptome analysis: Ferroptosis and potential inflammatory pathways in post-traumatic stress disorder

**DOI:** 10.3389/fpsyt.2022.841999

**Published:** 2022-10-05

**Authors:** Jie Zhu, Ye Zhang, Rong Ren, Larry D. Sanford, Xiangdong Tang

**Affiliations:** ^1^Sleep Medicine Center, Department of Respiratory and Critical Care Medicine, Mental Health Center, West China Hospital, Sichuan University, Chengdu, China; ^2^Sleep Research Laboratory, Center for Integrative Neuroscience and Inflammatory Diseases, Pathology and Anatomy, Eastern Virginia Medical School, Norfolk, VA, United States

**Keywords:** ferroptosis, post-traumatic stress disorder, transcriptome analysis, computational modeling, inflammatory pathways

## Abstract

**Background:**

Transcriptome-wide analysis of peripheral blood in post-traumatic stress disorder (PTSD) indicates widespread changes in immune-related pathways and function. Ferroptosis, an iron-dependent regulated cell death, is closely related to oxidative stress. However, little is known as to whether ferroptosis plays a role in PTSD.

**Methods:**

We conducted a comprehensive analysis of combined data from six independent peripheral blood transcriptional studies in the Gene Expression Omnibus (GEO) database, covering PTSD and control individuals. Differentially expressed genes (DEGs) were extracted by comparing PTSD patients with control individuals, from which 29 ferroptosis-related genes (FRGs) were cross-matched and obtained. The weighted gene co-expression network analysis (WGCNA), the Extreme Gradient Boosting (XGBoost) model with Bayesian Optimization, and the least absolute shrinkage and selection operator (LASSO) Cox regression were utilized to construct a PTSD prediction model. Single-sample Gene Set Enrichment Analysis (ssGSEA) and CIBERSORT revealed the disturbed immunologic state in PTSD high-risk patients.

**Results:**

Three crucial FRGs (ACSL4, ACO1, and GSS) were identified and used to establish a predictive model of PTSD. The receiver operating characteristic (ROC) curve verifies its risk prediction ability. Remarkably, ssGSEA and CIBERSORT demonstrated changes in cellular immunity and antigen presentation depending on the FRGs model.

**Conclusion:**

These findings collectively provide evidence that ferroptosis may change immune status in PTSD and be related to the occurrence of PTSD, which may help delineate mechanisms and discover treatment biomarkers for PTSD.

## Introduction

Post-traumatic stress disorder (PTSD) is a psychiatric syndrome involving the interaction of environments and genes. PTSD occurs after a traumatic experience and is followed by flashbacks, hallucinations, nightmares, constant alertness, and enhanced arousal (1). By definition, PTSD is associated with a traumatic event. However, data also suggest that the development of PTSD requires a genetic tendency that alters, to varying degrees, an individual’s response to, or recovery from, traumatic exposure (2).

The development of high-throughput sequencing technology has enabled unbiased identification of genes, pathways, and proteins related to PTSD pathophysiology. Data from five PTSD peripheral blood studies indicated that transcriptional disruption affects multiple immune-related pathways and molecules (3). In a review of similar studies, Heinzlemann and Gill concluded that PTSD develops as a result of altered epigenetic regulation and inflammatory genes that are highly active (4).

Despite the widespread observation of immune fluctuations, it remains unclear how specific mechanisms are activated or how key processes are regulated. On the other hand, the nervous system is particularly vulnerable to oxidative stress due to its high metabolic demands and dense composition of oxidation-sensitive lipid cells (5, 6). PTSD patients showed elevated serum lipid peroxidation and depleted antioxidant enzymes (7). Down-regulated expression of the antioxidant protease, superoxide dismutase (SOD), also was observed in PTSD patients (8).

Oxidative stress is a cellular state that occurs when the pro-oxidant molecules, such as reactive oxygen species (ROS), exceed the elimination power of the antioxidants (9). Antioxidant depletion leads to cell degeneration and apoptosis, making oxidative stress a primary molecular aging mechanism widely involved in multiple diseases.

In the presence of excess iron, or more precisely, the divalent ferrous ion Fe^2+^, can produce abundant ROS such as soluble hydroxyl radicals or lipid alkoxy radicals (10). Known as the Fenton reaction, this is the main source of ROS in the cell produced by Fe^2+^. By generating ROS, mitochondrial respiration is reduced, lipids are peroxidized, enzymes are oxidized, and neuronal damage is possible (11). Moreover, ROS and mitochondrial function seem to be closely related to the innate immune system. Mitochondria-derived ROS can trigger some inflammasomes such as nucleotide-binding and oligomerization domain (NOD)-like receptors (NLRs), and Melanoma (AIM) 2-like receptors (ALRs) (12, 13).

Another process closely related to iron metabolism and ROS is ferroptosis. Ferroptosis is a form of iron-dependent cell death induced by oxidative stress, and that involves molecular pathways common to oxidative stress, such as lipid peroxidation and glutathione (GSH) depletion (14). The Fenton reaction is the critical step of ferroptosis. A high level of iron produces excessive ROS and leads to liposome peroxidation, which leads to cell death. Although first found in cancer cells, ferroptosis has been linked to several neurological illnesses, such as Alzheimer’s, Parkinson’s, and stroke (15–17). Stefanovic et al. found lower GSH transferase levels in PTSD (18), suggesting that ferroptosis may be involved in the pathophysiological process of PTSD. These studies suggest that ferroptosis may be a key influence in the pathological processes of PTSD.

**FIGURE 1:**
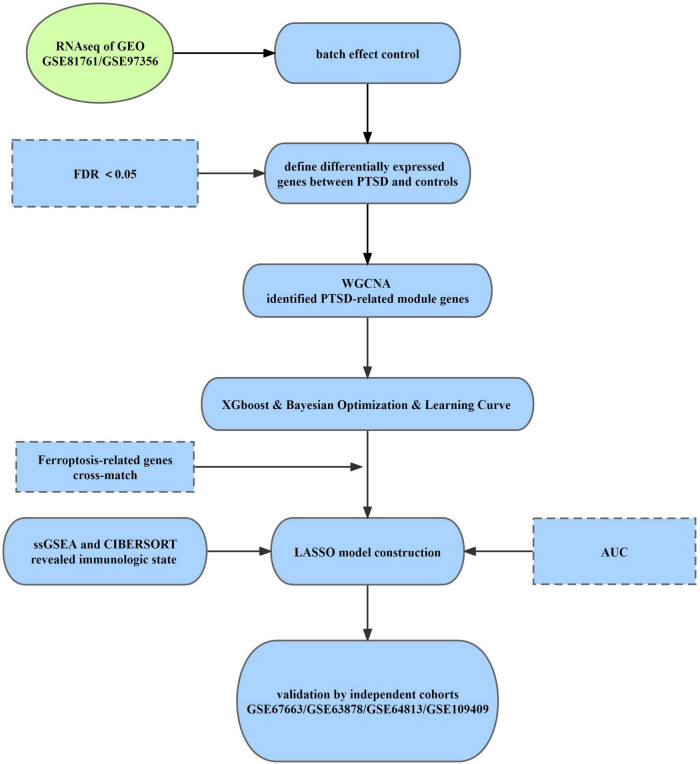
Workflow of the study.

The main aim of the current study is to synthesize available data from transcriptional studies of PTSD and to elucidate the association of ferroptosis-related genes (FRGs) with the pathophysiology of PTSD. Six independent studies from the Gene Expression Omnibus (GEO) database were included. Multiple algorithms were used in this study to establish a risk prediction model for PTSD, including the weighted gene co-expression network analysis (WGCNA), the Extreme Gradient Boosting (XGBoost) model with Bayesian Optimization, and the least absolute shrinkage and selection operator (LASSO) Cox regression (Figure 1). Furthermore, immune cell and function analyses were conducted to reveal the possible underlying mechanism of PTSD assessments.

## Materials and methods

### Data availability

The RNA sequencing (RNA-seq) data of PTSD patients were obtained from the GEO database (accession numbers: GSE97356, GSE81761, GSE63878, GSE64813, GSE67663, and GSE109409) (Table 1). GSE97356 contains 324 World Trade Center responders, of which 123 individuals are in the PTSD group, and 201 individuals are controls. GSE81761 includes military service members with PTSD (*n* = 39) and controls without PTSD (*n* = 27) at baseline. GSE63878 contains 96 samples from U.S. Marines deployed to conflict zones, half of whom returned with PTSD. GSE64813 involved 188 samples of service members, and half with PTSD. GSE67663 summarizes the gene expression profiles of 112 PTSD cases and 72 controls. GSE109409 contains 85 Canadian infantry soldiers, of whom 27 were positive for PTSD. All research projects used peripheral blood to obtain transcriptome-wide RNA-Seq data, and both are publicly available. Thus, the present study was exempt from requiring approval from local ethics committees. The GSE97356 and GSE81761 datasets were used as training sets, while the others were used as independent validation datasets. The ferroptosis-related genes (FRGs) list was derived from previously published research (19–23).

**TABLE 1:** Baseline of included datasets.

GSE97356	PTSD group (*N* = 123)	Control group (*N* = 201)
Age (mean)	52.5	51.4
**Race (*n*%)**		
Caucasian	102 (82.9%)	181(90.0%)
Other	21(17.1%)	20(9.9%)
Sex (*n*%)	Not applicable	Not applicable

**GSE81761**	**PTSD group (*N* = 39)**	**Control group (*N* = 27)**

Age (mean)	31.1	35.9
**Race (*n*%)**		
White	25 (64.1%)	20 (74.1%)
Non-white	14 (35.9%)	7 (25.9%)
**Sex (*n*%)**		
Male	37 (94.9%)	26 (96.3%)
Female	2 (5.1%)	1 (3.7%)

**GSE63878**	**PTSD group (*N* = 48)**	**Control group (*N* = 48)**

Age (mean)	22.2	22.4
**Race (*n*%)**		
Caucasian	26 (54.2%)	26 (54.2%)
African American	4 (8.3%)	4 (8.3%)
Native American Mexican	13 (27.1%)	13 (27.1%)
Asian and Other	5 (10.4%)	5 (10.4%)
**Sex (*n*%)**		
Male	48 (100%)	48 (100%)
Female	0 (0%)	0 (0%)

**GSE64813**	**PTSD group (*N* = 94)**	**Control group (*N* = 94)**

Age (mean)	23.1	23.4
**Race (*n*%)**		
Caucasian	52 (55.3%)	52 (55.3%)
African American	8 (8.5%)	8 (8.5%)
Native American Mexican	26 (27.7%)	26 (27.7%)
Asian and Other	10 (10.6%)	10 (10.6%)
**Sex (*n*%)**		
Male	94 (100%)	94 (100%)
Female	0 (0%)	0 (0%)

**GSE67663**	**PTSD group (*N* = 112)**	**Control group (*N* = 72)**

Age (mean)	41.9	43.3
**Race (*n*%)**		
African American	102 (91.1%)	71 (98.6%)
Others	10 (8.9%)	1 (1.4%)
**Sex (*n*%)**		
Male	25 (22.3%)	21 (29.2%)
Female	87(77.7%)	51 (70.8%)

**GSE109409**	**PTSD group (*N* = 27)**	**Control group (*N* = 58)**

Age (mean)	28.7	30.3
Race (*n*%)	Not applicable	Not applicable
Sex (*n*%)	Not applicable	Not applicable

### Combined transcriptional data-processing and batch effect control

All statistical analyses were conducted using the R program version 3.6.2 and GraphPad software (Prism 8). Each individual’s gene expression profiles were summarized after the microarray probes were mapped with gene symbols according to the chips and platforms. If multiple microarray probes were mapped to one single gene, the expression level was expressed as the mean value. The analysis did not include missing data or samples with low coverage. The gene expression values were log2-transformed, namely log2 Fold Change (log2FC), before normalization. The batch correction was conducted using the R package ComBat and sva functions to reduce cohort effects and remove system variability from technical, clinical, or demographic factors (3, 24). Subsequently, combined and normalized cohorts contained gene expression data from two GEO cohorts that included PTSD and control individuals. We performed a principal component analysis (PCA) to verify whether the batch effect was eliminated. Continuous variables were compared between groups using the equal-variance *T*-test. Unless otherwise noted, the significance threshold for the *P*-value was set to 0.05.

### Weighted correlation network analysis

The Wilcoxon test for non-parametric distributions was performed to detect differential gene expressions (DGEs) between PTSD and controls samples by the limma package. The weighted gene co-expression network analysis (WGCNA) is a common method to transform gene expression data into a co-expression network and identify disease-related gene modules and key genes affecting phenotypic traits (25, 26). The R program’s WGCNA package was utilized on DGEs data to identify highly connected modules, which summarized specific gene expression patterns related to PTSD. Under the proper soft threshold power, clustering analysis can successfully establish a standard scale-free network, and then overlapping WGCNA function was used to get a Topological Overlap Matrix (TOM). Similar modules were merged by the hierarchical clustering method with a height cut-off of 0.25. Module eigengenes (MEs) were principal components and summarized all gene expression patterns into a specific module. Subsequently, module-trait associations were estimated using spearman’s correlation analysis (in our study, clinical trait refers to PTSD). The module with the highest spearman’s correlation coefficient was extracted. Genes clustered in the module genes were then cross-matched with FRGs, thus identifying FRGs potentially crucial in PTSD development.

### Hyperparameter optimization and feature importance ranking

In order to further refine the screening of key genes, we employed the Extreme Gradient Boosting (XGBoost) algorithm. The XGBoost algorithm excels as a method for combining multiple learning algorithms into one superior predictive algorithm. It consists primarily of two parts: a decision tree algorithm and a gradient boosting algorithm (27). Boosting is accomplished by setting up weak evaluators individually and integrating multiple weak evaluators iteratively. Because hyperparameters can greatly impact the classification performance of the XGBoost model, the Bayesian parameter optimization based on Gaussian processes was applied as a way to adjust them (28). Four main hyperparameters were associated with the Bayesian optimization in this article: Eta (Learning rate), Max depth (Maximum depth of a tree), Min child weight (Minimum sum of instance weight needed in a child), and Subsample (Subsample ratio of the training instances). We used the area under the curve (AUC) as the objective function. Ranks of features were determined by the average gain of each feature across all trees. High-value features can be considered more significant for prediction than low-value features. An analysis of the model’s performance was compared according to the learning curve. As a result, the classification model’s generalization ability (overfitting or underfitting) could be effectively evaluated (29).

### Functional annotation and protein-protein interaction networks

Gene Ontology (GO) enrichment and Kyoto Encyclopedia of Genes and Genomes (KEGG) pathway analyses were conducted to better understand the biologic function of DGEs and FRGs. GO analysis utilized the Biological Process term, which provides current scientific information about the functions of encoding and non-coding genes and allows exploring how individual genes contribute to an organism’s biology at the molecular, cellular, and organism levels. KEGG database provides information for biological system functions such as cells, organisms, and ecosystems, mainly generated from large-scale datasets produced by genome sequencing and other high-throughput technologies. The intersection of DGEs and FRGs was assessed via the R VennDiagram package. The GO Biological Process and KEGG pathway analyses identified major biological terms via the R “clusterProfiler” package. The R “GOplot” package was employed to visualize the enrichment terms.

The Search Tool for the Retrieval of Interacting Genes database^1^ provides protein interaction information from large-scale sequencing sources (30). Using this tool, the physical and functional associations among specific gene clusters (based on user requirements) can be computationally predicted. A protein–protein interaction (PPI) network among intersection genes of DGEs and FRGs was calculated by topology analysis using Maximal Clique Centrality (MCC).

### Establishment of the risk prediction model

Genome-wide analysis of gene expression levels and high throughput technology produces a large amount of data that allows statistical analyses of complex diseases’ genetic causes. Regularization via the least absolute shrinkage and selection operator (LASSO) is often used to reduce the selected set of explanatory variables in examining the associations between all biomarkers and a given phenotype (31). The LASSO model construction was accomplished using the R package “glmnet.” After tenfold cross-validation with minimum standards to determine the penalty parameters (λ), the Lasso model was established. The Receiver Operating Characteristic (ROC) curve verified the prediction ability for the risk of illness.

### Gene set enrichment derived from immune cell markers and CIBERSORT analysis

Single-sample Gene Set Enrichment Analysis (ssGSEA) classified gene sets with immune biological roles by identified immune markers (32, 33). The immune markers comprised 782 immune-related genes representing diverse immunologic cells and functions (34). The expression data were changed into ssGSEA scores to predict the abundance of each gene set type in individual samples. The gene expression profile of two combined PTSD studies was transformed into a gene set enrichment profile.

Cell-type identification by estimating relative subsets of RNA transcript, also named CIBERSORT, is a computational algorithm that distinguishes 22 immune cell types retrieved from RNA-sequencing gene expression profiles (35). Cell types including B cells naïve, B cells memory, Plasma cells, T cells CD8, T cells CD4 naïve, T cells CD4 memory resting, T cells CD4 memory activated, T cells follicular helper, T cells regulatory (Tregs), T cells gamma delta, NK cells resting, NK cells activated, Monocytes, Macrophages M0, Macrophages M1, Macrophages M2, Dendritic cells resting, Dendritic cells activated, Mast cells resting, Mast cells activated, Eosinophils, and Neutrophils were estimated in each sample. And in that way, the gene expression profile was transformed into an immune cell profile.

## Results

### Differentially expressed genes and ferroptosis-related genes in the combined gene expression omnibus cohort

**FIGURE 2:**
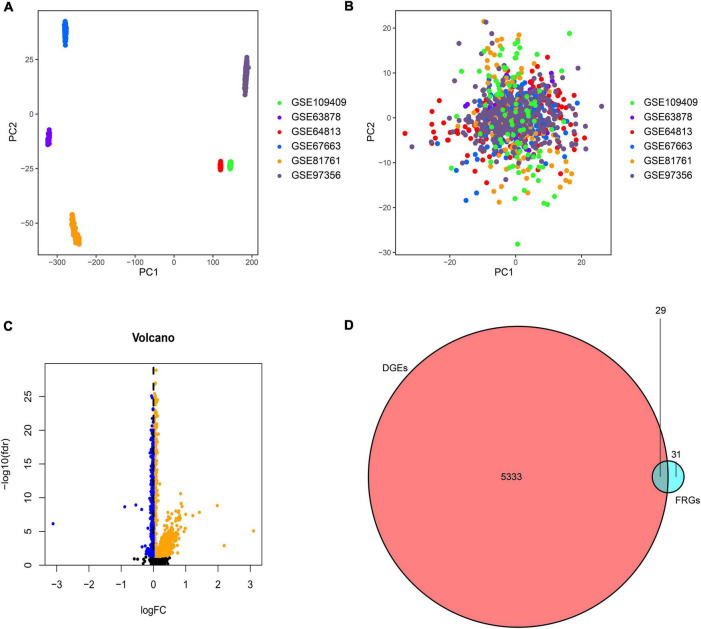
Principal component analysis (PCA) of the six GEO datasets. **(A)** Scatter plots present the samples based on two principal components (PC1 and PC2) without removing the batch effect. **(B)** Scatter plots to present the samples with the removal of batch effect. **(C)** Volcano plot of DGEs in the combined GEO cohort. The orange spots represented the up-regulated genes, and the blue spots represented the down-regulated genes between PTSD and controls. **(D)** Venn diagram of DGEs and FRGs showed the cross-match gene set contained 29 crucial FRGs.

The samples based on the unnormalized expression values showed a distribution bias by batch (Figure 2A). After normalization, the PCA plot indicated that the batch effect was removed from the different platforms (Figure 2B), and 185 PTSD subjects and 248 controls were included in the training datasets. The batch correction had a significant impact on the log2FC value of the differential genes. The absolute value of average log2FC decreased from 89 to less than 1. Therefore, instead of utilizing log2FC, we used a stronger *P*-value criterion, namely the *P*-value after FDR (false discovery rate) correction. A total of 5362 DGEs were screened from 19,281 genes at baseline using an FDR value less than 0.05, of which 369 were up-regulated, and 4993 were down-regulated (Figure 2C). After matching with 60 FRGs reported in previous studies, we obtained 29 differential FRGs (Figure 2D): ACACA, ACO1, ACSF2, ACSL3, ACSL4, AIFM2, AKR1C3, ALOX5, ALOX12, ALOX15, CBS, CD44, CHAC1, CISD1, CRYAB, CS, DPP4, EMC2, FADS2, FANCD2, FDFT1, FTH1, G6PD, GCLC, GCLM, GLS2, GOT1, GPX4, and GSS.

### Functional annotation of the differential ferroptosis-related genes

**FIGURE 3:**
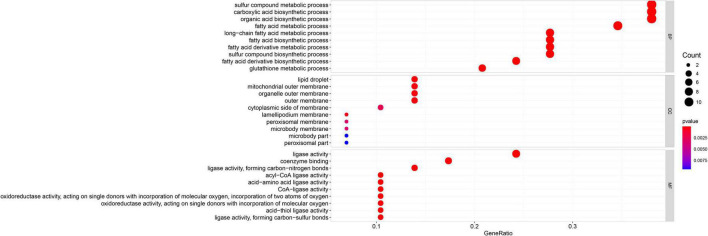
Gene Ontology enrichment of 29 differential FRGs. The count shows the number of genes enriched in the pathway. Gene ratio: the ratio of the FRGs number in this term to the total number of FRGs.

**FIGURE 4:**
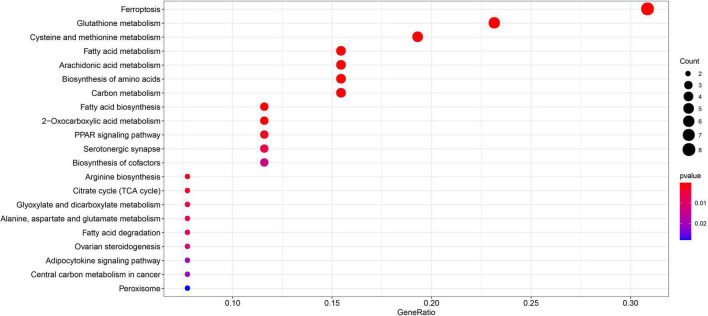
Kyoto encyclopedia of genes and genomes enrichment of 29 differential FRGs. The count shows the number of genes enriched in the pathway. Gene ratio: the ratio of the FRGs number in this term to the total number of FRGs.

Gene Ontology enrichment analysis related to biological processes (BP) found that the 29 differential FRGs were enriched in several metabolic pathways, including carboxylic acid biosynthesis, organic acid biosynthesis, long-chain fatty acid metabolism, and glutathione metabolism. Cellular components (CC) genes were concentrated in various organelle membranes, e.g., organelle outer membrane, mitochondrial outer membrane, and microbody membrane. Molecular function (MF) genes were mainly enriched in terms of the activity of multiple enzymes, including acyl-CoA ligase activity, acid-amino acid ligase activity, and acid-thiol ligase activity (Figure 3). Not surprisingly, in the KEGG pathway analyses, the 29 differential FRGs were notably associated with ferroptosis and some metabolic pathways similar to those revealed by GO enrichment, such as fatty acid biosynthesis, 2-Oxocarboxylic acid metabolism, and glutathione metabolism (Figure 4).

### Construction and analysis of protein–protein interaction network

**FIGURE 5:**
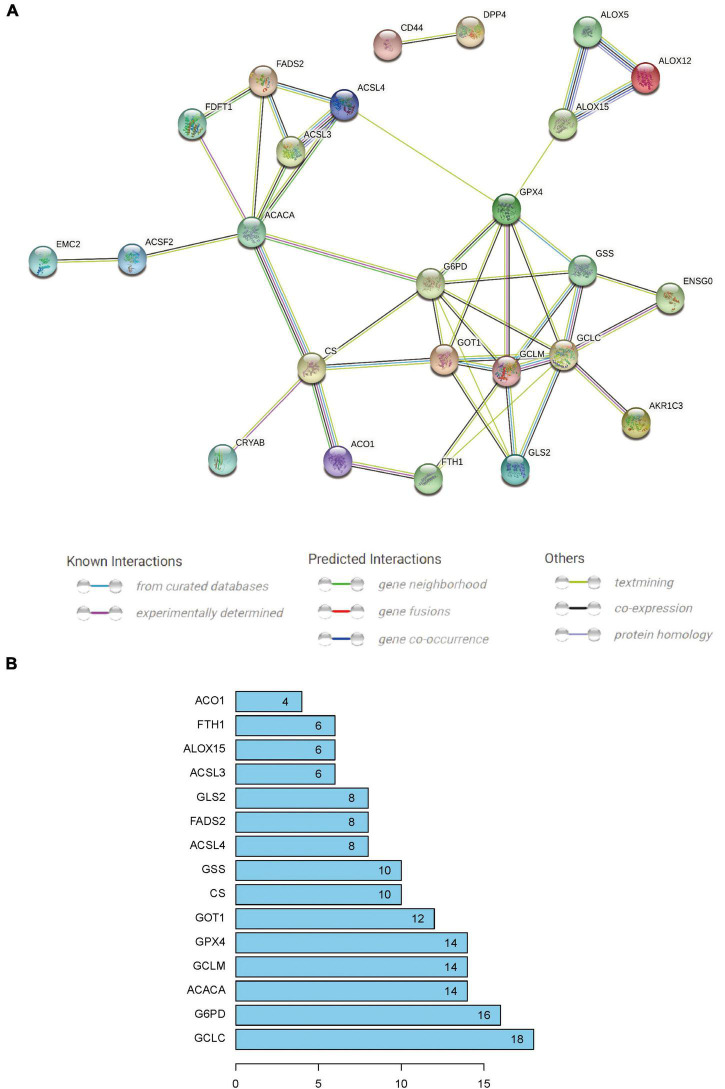
**(A)** Protein–protein interaction network generated by STRING database analysis indicating direct and indirect associations among 29 crucial FRGs. **(B)** Fifteen significant nodes of the PPI network were screened using an interaction score > 0.4; these were the most widely connected and are sorted by the number of connected nodes.

The PPI network was constructed with the 29 differential FRGs using the STRING database (Figure 5A). Subsequently, 15 significant network nodes (GCLC, G6PD, ACACA, GCLM, GPX4, GOT1, CS, GSS, ACSL4, FADS2, GLS2, ACSL3, ALOX15, FTH1, ACO1) were identified with a PPI combined score > 0.4, which indicated a medium to high confidence network (Figure 5B).

### Co-expression network construction

**FIGURE 6:**
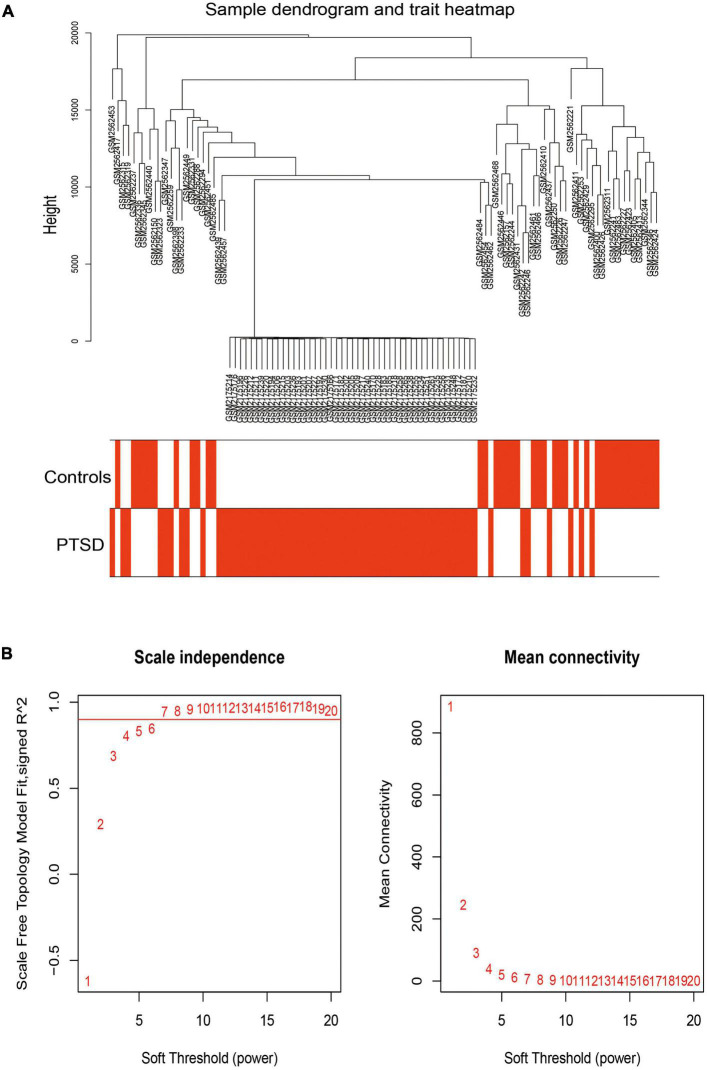
**(A)** The gene hierarchy clustering plots showed that all samples were divided into two clusters (in the red and white plot) with the WGCNA algorithm method. **(B)** A scale-free network distribution was with stable average connectivity when the soft threshold power β was set to 7.

**FIGURE 7:**
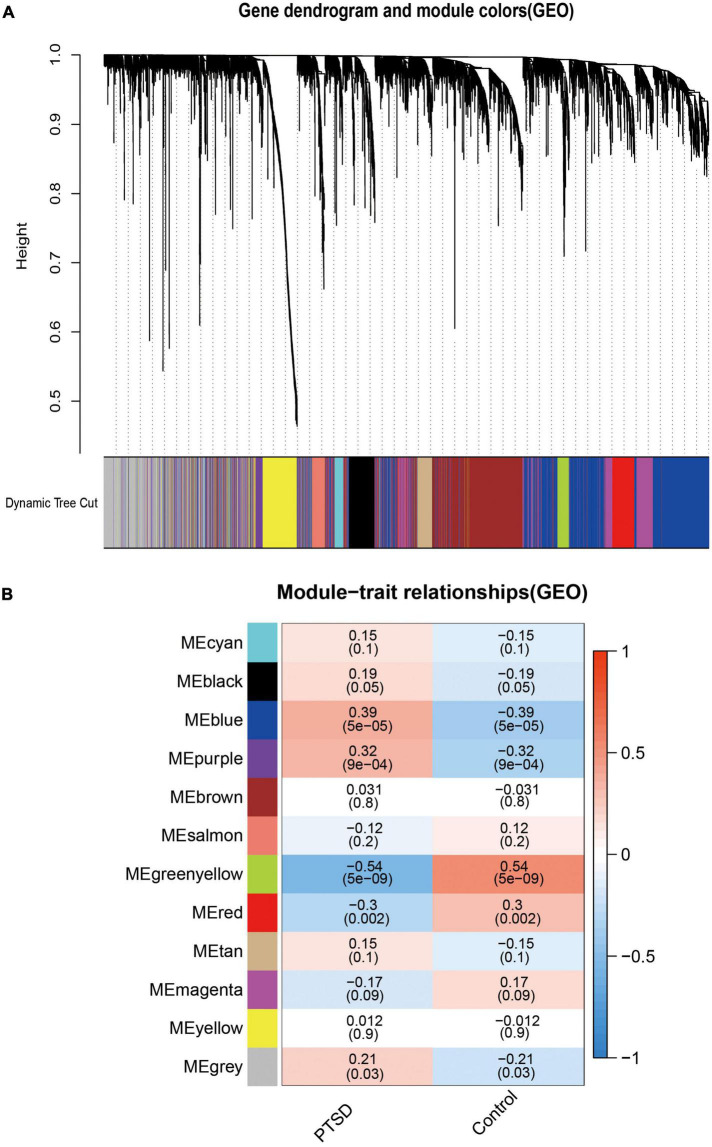
**(A)** Through the hierarchical clustering method, similar modules were clustered in 12 module eigengenes (MEs), which summarized all gene expression patterns into a specific module. **(B)** Spearman’s correlation analysis between the MEs and PTSD indicated that the most relevant object module was MEgreenyellow (correlation coefficient = −0.54, *P* = 5e-09).

We used WGCNA to assess highly connected modules by integrating DGEs of PTSD cases compared to all control individuals. The gene hierarchy clustering plots showed two clusters in PTSD patients and controls by the WGCNA algorithm method (Figure 6A). Outliers in height above 20,000 were removed. When the soft threshold power β was set to 7, the scale-free Topology fitting index R^2 was greater than 0.9 and the mean connectivity was stabilized, indicating a good network connection (Figure 6B). After removing highly similar modules (Figure 7A), 12 gene cluster modules (Figure 7B) were generated as MEcyan (96 genes), MEblack (258 genes), MEblue (1550 genes), MEpurple (173 genes), MEbrown (989 genes), MEsalmon (135 genes), MEgreenyellow (149 genes), MEred (274 genes), MEtan (149 genes), MEmagenta (434 genes), MEyellow (385 genes), and MEgray (770 genes). The spearman’s correlation analysis revealed each module’s correlation coefficient with PTSD. We chose the module with the largest correlation coefficient, MEgreenyellow (correlation coefficient = -0.54, *P* = 5e-09), as the critical module for further analysis.

### Functional annotation of the MEgreenyellow module genes

**FIGURE 8:**
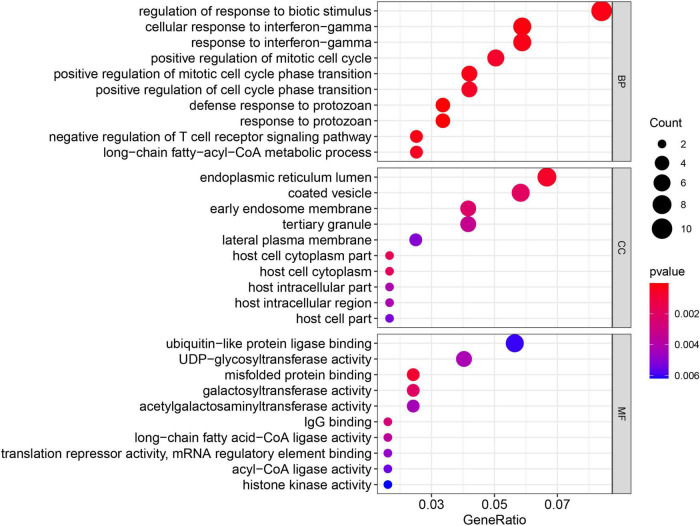
Gene Ontology enrichment of 149 MEgreenyellow genes. The count shows the number of genes enriched in the pathway. Gene ratio: the ratio of the gene number in this term to the total number of the MEgreenyellow module.

**FIGURE 9:**
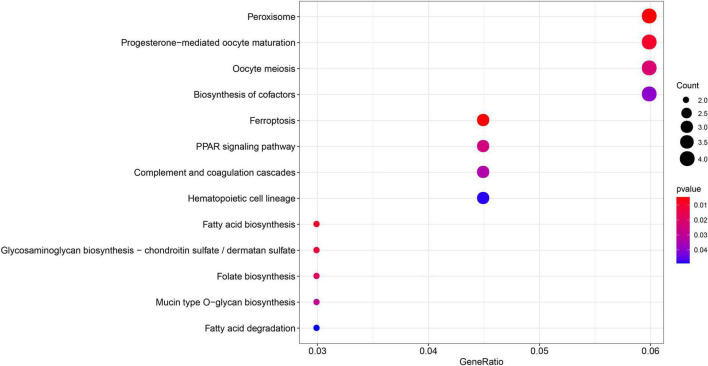
Kyoto encyclopedia of genes and genomes enrichment of 149 MEgreenyellow genes. The count shows the number of genes enriched in the pathway. Gene ratio: the ratio of the gene number in this term to the total number of the MEgreenyellow module.

The GO enrichment and KEGG pathway analyses were performed again for the 149 MEgreenyellow genes (Figures 8, 9). For GO analysis, these genes aggregated in multiple immune-related responses, such as cellular response to interferon-gamma, negative regulation of T cell receptor signaling pathway, and IgG binding. It is worth noting that the 149 MEgreenyellow genes were also enriched in ferroptosis in the KEGG pathway and were involved in some similar metabolic pathways, including long-chain fatty acid-CoA ligase activity and fatty acid biosynthesis.

### Key genes assessed by XGBoost

**FIGURE 10:**
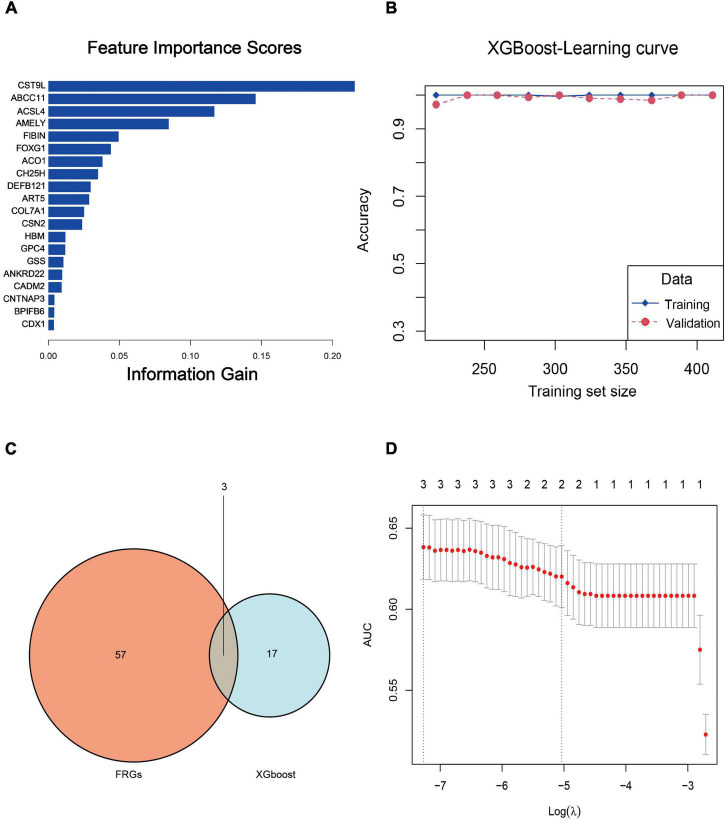
**(A)** Feature importance rankings for the top 20 genes identified by the XGBoost model. **(B)** Accuracy rates in the training and validating processes upon the learning curve. **(C)** Venn diagram of the top 20 genes and FRGs. After cross-matching 20 XGBoost genes and 60 FRGs, we identified three crucial FRGs related to PTSD development, which were ACSL4, ACO1, and GSS. **(D)** LASSO coefficient profile plot of the three crucial FRGs plotted against the log (lambda) sequence.

Table 2 shows parameter ranges and optimized values for the XGBoost model. Accordingly, we obtained the optimal feature subset and the hyperparameter combination that provided the highest AUC. In the learning curve, the optimal model satisfied both the accuracy of the training set and the validation set at the same time (Figure 10B). Based on the rank order of each feature in the XGBoost model, the top 20 key genes were retained (Figure 10A).

**TABLE 2:** Main hyper-parameter range and optimized value.

Hyperparameter	Range	Optimized value
Eta	(0.01, 0.046)	0.03577263
Max depth	(6, 8)	8
Min child weight	(1, 9)	2
Subsample	(0.5, 0.8)	0.6688349

### The least absolute shrinkage and selection operator model construction

**FIGURE 11:**
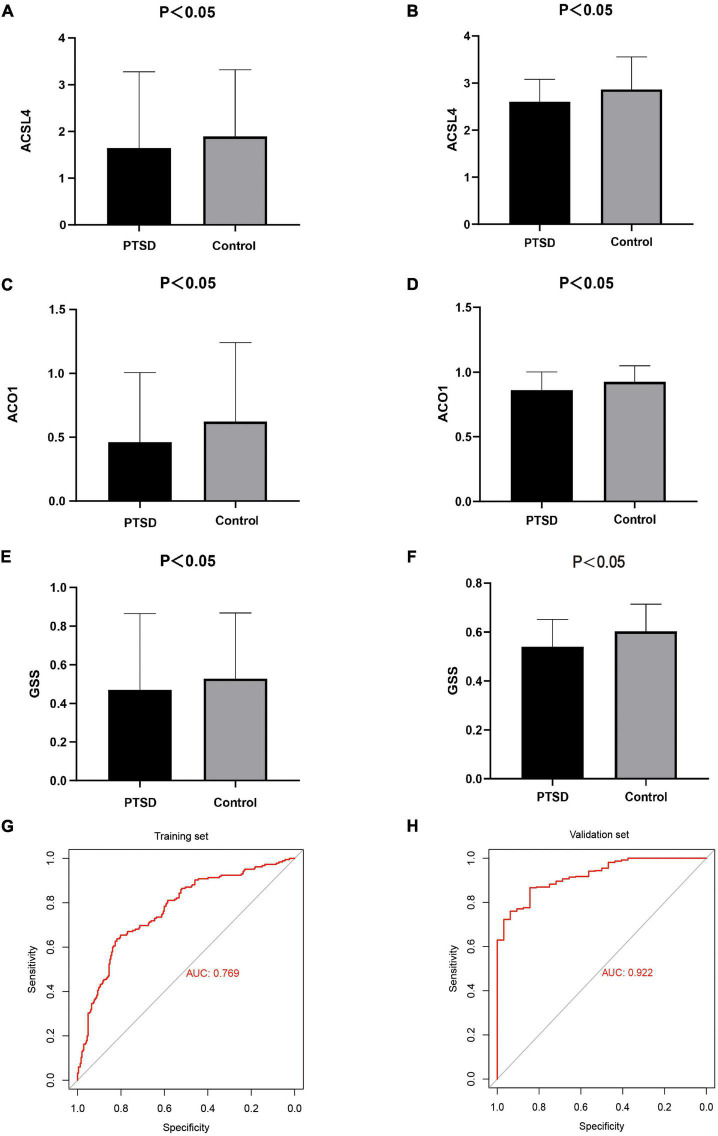
**(A,B)** *t*-Tests found that ACSL4 was down-expressed in the training datasets and the validation datasets. **(C,D)** ACO1 was down-expressed in the training datasets and the validation datasets. **(E,F)** GSS was down-expressed in the training datasets and the validation datasets. **(G,H)** The AUC of ROC curves reached 0.769 and 0.922 in the training datasets and the validation datasets, indicating the LASSO model has good diagnostic accuracy.

After cross-matching 60 FRGs and 20 key genes, we identified three crucial FRGs related to PTSD development: ACSL4, ACO1, and GSS (Figure 10C). They were also significant nodes of our PPI network. *t*-Tests showed that all three genes were down-expressed in the training datasets and the validation datasets (Figures 11A–F) compared with control individuals. The LASSO model of PTSD was constructed using the three intersecting genes. As shown in Figure 10D, the optimal value of λ was set when a 3-FRG signature was generated as follows: estimation score = ACSL4 × 0.000296 + ACO1 × –0.001032 + GSS × 0.001216. The PTSD group received a higher score than the control group (*P* < 0.001). ROC curves evaluated the estimation score’s predictive performance for PTSD, and the AUC reached 0.769 in the training datasets and 0.922 in the validation datasets (Figures 11G,H).

### Relationship between immune status and estimation risk of post-traumatic stress disorder

**FIGURE 12:**
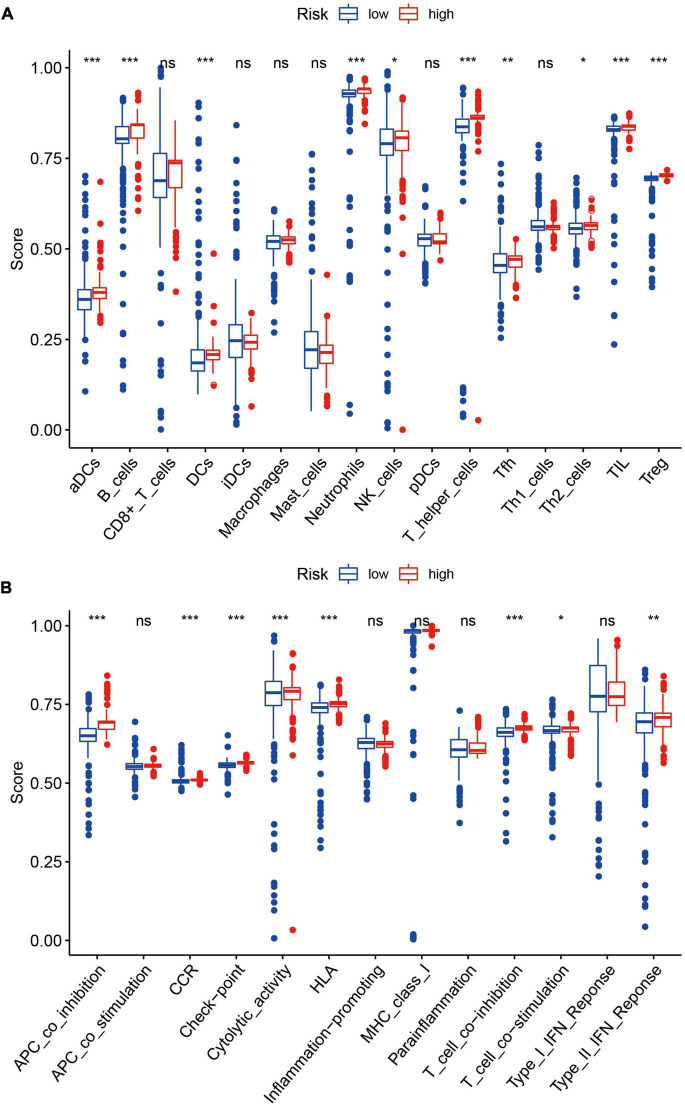
**(A)** Elements related to antigen presentation process contents such as aDCs, B cells, and DCs were significantly up-regulated in the high-risk group (all *P* < 0.05). Elements related to cellular immunity, such as neutrophils, NK cells, T helper cells, Tfh, Th2 Cells, TIL, and Tregs, were also up-regulated in the high-risk group. *P < 0.05; **P < 0.01; ***P < 0.001. **(B)** The contents of the antigen presentation process, including APC co-inhibition, CCR, Check-point, Cytolytic activity, HLA, T cell co-inhibition, T cell co-stimulation, and Type II IFN Response were significantly up-regulated in the high-risk group. *P < 0.05; **P < 0.01; ***P < 0.001.

We quantified the ssGSEA enrichment scores of each sample and took the median value of the estimation score as the threshold to divide the high and low-risk groups. As shown in Figure 12A, elements related to antigen presentation process contents such as aDCs (activated dendritic cells), B cells, and DCs (dendritic cells) were significantly up-regulated in the high-risk group. Elements related to cellular immunity, such as neutrophils, T helper cells, NK cells, Tfh (follicular helper T cells), Th2 Cells, TIL (tumor-infiltrating lymphocytes), and Tregs (regulatory T cells), were up-regulated in the high-risk group. The antigen presentation process includes APC (antigen-presenting cell) co-inhibition, CCR (CC chemokine receptor), Check-point, Cytolytic activity, HLA (human leukocyte antigen), T cell co-inhibition, T cell co-stimulation, and Type II IFN Response were also up-regulated in the high-risk group (Figure 12B). The high-risk group showed elevated levels in cellular immunity and antigen presentation function, which may be associated with disturbances in ferroptosis.

**FIGURE 13:**
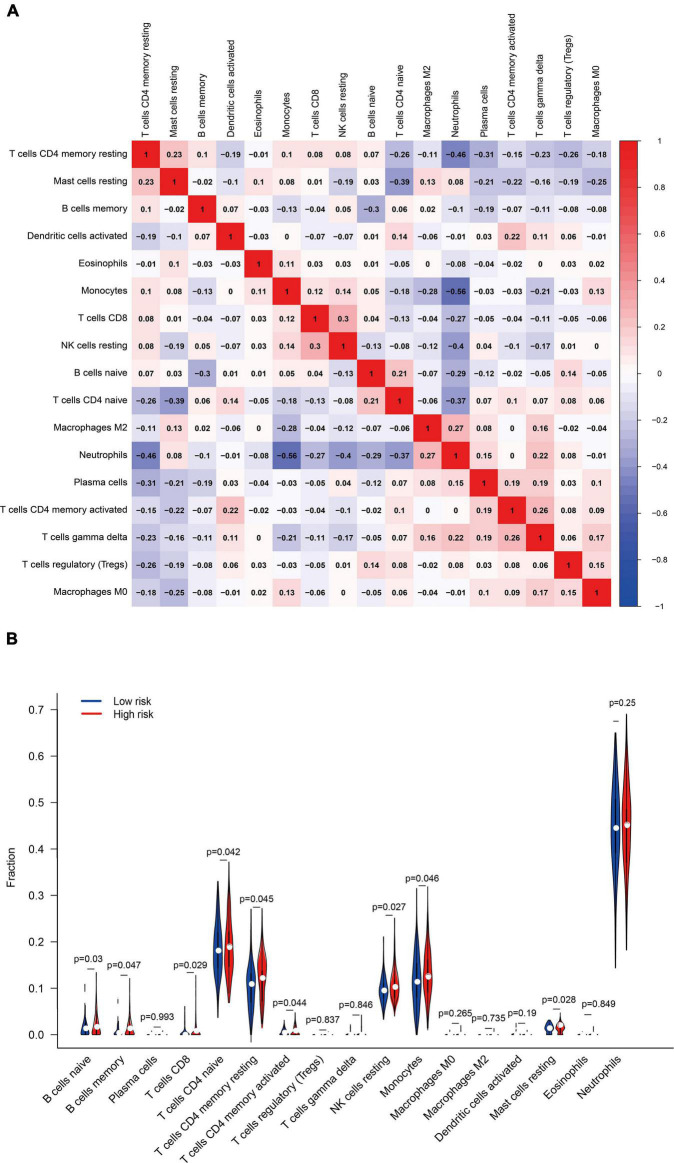
**(A)** Pearson correlation coefficient revealed the correlation between 17 immune cells. **(B)** The differential expression of immune cells in the high- and low-risk groups.

The abundance of 22 immune cells in each sample was compared between the high- and low-risk groups. Pearson correlation coefficient was used to calculate the correlation between components. T cells follicular helper, NK cells activated, Macrophages M1, Dendritic cells resting, and Mast cells activated were excluded because they were present in zero amounts in each sample. Figure 13A displayed the correlation among the above 17 immune cell types. A total of four immune cell types were obviously correlated. Neutrophils negatively related to T cells CD4 memory resting (*r* = –0.46), Monocytes (*r* = –0.56), and NK cells resting (*r* = –0.40), suggesting that there may be an antagonistic relationship between neutrophils, T cells CD4 memory resting, Monocytes, and NK cells resting.

The differential expression of immune cells assessed by CIBERSORT was shown in Figure 13B. B cells naïve, B cells memory, T cells CD8, T cells CD4 naïve, T cells CD4 memory resting, T cells CD4 memory activated, NK cells resting, Monocytes, and Mast cells resting were up-regulated in the high-risk group, taking *P* < 0.05 as the threshold. These results also confirmed the ssGSEA enrichment result, indicating that ferroptosis is involved in the disorder of the immune state in PTSD patients. FRGs are potential indicators to evaluate the PTSD risk and underlying immune status.

## Discussion

We conducted a comprehensive transcriptome-wide analysis covering PTSD cases and control individuals by combining six independent research datasets, intending to reveal the potential involvement of FRGs in the pathophysiology of PTSD. As the first step of the study, we applied batch normalization to reduce the batch effect, allowing us to improve statistical capabilities and explicitly validate different molecular pathways in PTSD. The enrichment analysis of GO and KEGG suggested a direct relationship with glutathione metabolism, which is a critical process in ferroptosis (36). GSH is an essential intracellular antioxidant against oxidative stress (19, 37) synthesized from glutamate, cysteine, and glycine (19). Glutamate accumulation in oxidative stress inhibits the import of cysteine, resulting in GSH depletion and lipid peroxide accumulation (38, 39).

Dixon et al. first described ferroptosis (40), which was subsequently defined as iron-dependent regulated necrosis accompanied by lipid peroxidation (19). The main biochemical mechanism of ferroptosis is the catalysis of polyunsaturated fatty acids (PUFAs) that causes lipid peroxidation under the action of catalytic Fe (II) (41). Animal models of PTSD suggest increased iron in cognition-related brain regions, resulting in neuronal injury (42). Hence, high catalytic Fe (II) abundance indicates high levels of oxidative stress. PUFAs are frequently oxidized by lipoxygenases and reduced by the enzyme glutathione peroxidase 4 (GPX4) and its cofactor, GSH (20, 43). Intracellular GSH is synthesized from cysteine. Thus cysteine depletion leads to intracellular GSH exhaustion and triggers ferroptosis (40), indicating that maintaining certain cysteine levels is critical for protecting cells from ferroptosis. The requirement for cysteine for protection from ferroptosis is related to the activity of GPX4 (38, 44). Therefore, the inhibition of GPX4 and depletion of GSH results in elevated lipid peroxides and cell death induced by ferroptosis (19, 40, 43). Our study identified three crucial genes predictive for the risk of developing PTSD, which are also important components of ferroptosis. ACSL4, ACO1, and GSS regulate lipid, iron, cysteine, and glutathione metabolic processes, which participate in the complex biological interplay of ferroptosis (45).

ACSL4 (Acyl-CoA ligase 4) encodes an isozyme of the long-chain fatty-acid-coenzyme A ligase family, thereby exerting significant effects in lipid biosynthesis (46). ACSL4 helps produce arachidonic acid (AA) or adrenic acid (AdA) containing phosphatidylethanolamine, which is involved in lipid peroxidation for ferroptosis (47, 48). ACSL4 activation contributes to ferroptosis-induced brain injury and neuroinflammation in ischemic stroke (49). ACO1 (Aconitase 1) encodes an essential enzyme that can regulate iron levels inside cells, and knockdown of ACO1 can suppress ferroptosis induced by amino acid/cysteine deprivation (20, 50). Intracellular iron and ACO1 expression were found to engage in a directional cross-talk relationship in adipose tissue, simultaneously affecting its adipogenic capacity and connecting iron metabolism and adipogenesis (51). GSS (Glutathione Synthetase) is the core gene that affects glutathione synthesis and metabolism (52). Mutations in GSS cause glutathione synthetase deficiency and result in various metabolic diseases (53–55).

The enrichment analysis of highly connected module genes in WGCNA revealed immune response pathways, including the cellular response to interferon-gamma, negative regulation of T cell receptor signaling pathway, and IgG binding. Meanwhile, the ssGSEA and CIBERSORT analysis based on the FRGs model revealed specific immune status differences between risk groups, particularly in cellular immunity and antigen presentation, which primarily were up-regulated in the high-risk group. Our result is consistent with recent research that reported direct or indirect relationships between the immune response and PTSD. For example, combined data analysis extracted from five transcriptome studies found perturbed gene expression in aggregated inflammatory pathways, including cytokine, innate immune, and type I interferon (3). Immune responses are up-regulated in PTSD at baseline and down-regulated after symptom improvement (1). The pro-inflammatory cytokines in peripheral blood cells were examined, revealing that increased CRP, IL-6, TNF-α, IL-1β, and IFN-γ were related to PTSD symptoms (56). Transcriptional sequencing of peripheral blood from PTSD patients also supported roles for innate immune and interferon signaling genes in developing the pathophysiology underlying PTSD (57).

Recent work indicates that ferroptosis-related cell death is a potent activator of the innate immune system (36). Ruptured ferroptosis cells may release pro-inflammatory factors, such as damage-associated molecular patterns (DAMPs) (36), an immunogenic process that can increase the secretion of numerous proinflammation cytokines (58, 59). Moreover, ROS and oxidized lipoproteins are also key components of DAMPs. DAMPs stimulate inflammation by binding to pattern recognition receptors (PPRs), such as Toll-like receptors (TLRs), NLR families and the ALR families (12, 60). Activating these receptors further increase inflammatory responses by recruiting immune cells. Some ferroptosis cells release signals, such as PGE2, which could impact the local immune environment (44). Immunotherapy-activated CD8 + T cells have been found to promote ferroptosis-specific lipid peroxidation, which increased the efficacy of antitumor therapy in tumor diseases (61). It is thus reasonable to assume that ferroptosis could be a potential regulatory pathway in the immune changes associated with PTSD, making it a potential marker that could aid in recognizing the development of PTSD and treatment target for the disorder.

### Limitations

There are some limitations to our study. First, our model was established with a public database with a limited scale. Research across multiple centers will be required to verify our findings and assess their clinical utility. Second, a single hallmark to estimate the risk of PTSD development is insufficient because, as we know, the occurrence of PTSD is also related to a variety of environmental factors. The lack of time complexity is another limitation of our study. Due to the nature of the original data, cross-sectional studies were used to analyze the results. Further research is required to determine the validity of our conclusions in prospective studies. Our study suggests a potential role of ferroptosis in PTSD, even suggesting that it may serve as a therapeutic target for the treatment of PTSD. However, it should be emphasized that the link between ferroptosis and PTSD needs to be experimentally determined.

## Conclusion

In summary, our study defined a novel model associated with PTSD. The present work also indicated the potential immunological effect of ferroptosis in PTSD occurrence. Further investigation is needed to understand the mechanisms linking ferroptosis and the development of PTSD.

## Data availability statement

This data can be found here: Public datasets were available in the GEO database (https://www.ncbi.nlm.nih.gov/geo/). The corresponding accession numbers were given in the main text.

## Author contributions

JZ generated the original concept and performed the statistical analysis. YZ and RR helped writing the first draft of the manuscript. LS revised the entire manuscript. LS and XT supervised the entire study. All authors had full access to all study data and analyses, participated in preparing this report, and approved of its final, submitted form.

## Footnotes

1
http://string-db.org/
